# Targeting ERK induced cell death and p53/ROS-dependent protective autophagy in colorectal cancer

**DOI:** 10.1038/s41420-021-00677-9

**Published:** 2021-12-04

**Authors:** Wunan Mi, Chuyue Wang, Guang Luo, Jiehan Li, Yizheng Zhang, Meimei Jiang, Chuchu Zhang, Nannan Liu, Xinxiu Jiang, Ge Yang, Lingling Zhang, Ge Zhang, Yingjie Zhang, Yang Fu

**Affiliations:** 1 Department of Gastrointestinal Surgery, The First Affiliated Hospital of Zhengzhou University, 450052 Zhengzhou, China; 2 College of Biology, Hunan University, 410082 Changsha, China; 3 School of Biomedical Sciences, Hunan University, 410082 Changsha, China; 4 Department of Laboratory Medicine, The Third Xiangya Hospital, Central South University, 410013 Changsha, China; 5 Department of Ophthalmology, The First Affiliated Hospital of Zhengzhou University, 450052 Zhengzhou, China; 6 The Collaborative Innovation Center of Henan Province for Cancer Chemoprevention, 450052 Zhengzhou, China

**Keywords:** Colon cancer, Macroautophagy

## Abstract

In recent years, many studies have shown that autophagy plays a vital role in the resistance of tumor chemotherapy. However, the interaction between autophagy and cell death has not yet been clarified. In this study, a new specific ERK inhibitor CC90003 was found to suppress colorectal cancer growth by inducing cell death both in vitro and in vivo. Studies have confirmed that higher concentrations of ROS leads to autophagy or cell death. In this research, the role of CC90003-induced ROS was verified. But after inhibiting ROS by two kinds of ROS inhibitors NAC and SFN, the autophagy induced by CC90003 decreased, while cell death strengthened. In parallel, protective autophagy was also induced, while in a p53-dependent manner. After silencing p53 or using the p53 inhibitor PFTα, the autophagy induced by CC90003 was weakened and the rate of cell death increases. Therefore, we confirmed that CC90003 could induce autophagy by activating ROS/p53. Furthermore, in the xenograft mouse model, the effect was obtained remarkably in the combinational treatment group of CC90003 plus CQ, comparing with that of the single treatment groups. In a word, our results demonstrated that targeting ERK leads to cell death and p53/ROS-dependent protective autophagy simultaneously in colorectal cancer, which offers new potential targets for clinical therapy.

## Introduction

Colorectal cancer (CRC) is the fourth leading cause of cancer death in the world, with high morbidity and mortality [1, 2]. Statistics showed that 30–50% of patients with colorectal cancer will relapse after surgery [3]. Therefore, there is an urgent need to find a better treatment for CRC.

Autophagy, which is a metabolic process of orderly degradation and reuses [4], is considered to be the degradation of intracellular components by lysosomes [5–7]. In addition, the relationship between ERK and autophagy still remains unclear [8–10]. Moreover, how ERK-mediated autophagy played in the cell death is inconclusive [8, 11, 12]. Hence, we carried out deeper study.

Reactive oxygen species (ROS) is a byproduct of cellular oxygen metabolism and an indispensable signal factor for normal cells and tumor cells [13, 14]. In addition, studies have shown that the production of ROS is related to the induction of autophagy [15–17].

The tumor suppressor p53 is a key transcription factor that can regulate various cell functions, including cell cycle arrest, cell senescence, and apoptosis [18]. P53 plays a vital role in tumorigenesis and is also one of the most widely studied tumor suppressor genes [19]. In recent years, many studies have claimed that p53 may be involved in the process of autophagy [20, 21]. The p53 in cytoplasm can inhibit autophagy, and p53 accumulated in the nucleus can activate autophagy by interacting with a variety of targets. Therefore, p53 may be used as a new autophagy factor [22].

So, in the present study, a novel ERK inhibitor, CC90003, was used in the combined treatment with CQ. We also illustrated the relationship between cell death and autophagy in CRC and explored the role of p53 and ROS in it.

## Results

### Targeting ERK inhibited colorectal cancer growth and induced apoptosis

**Fig. 1:**
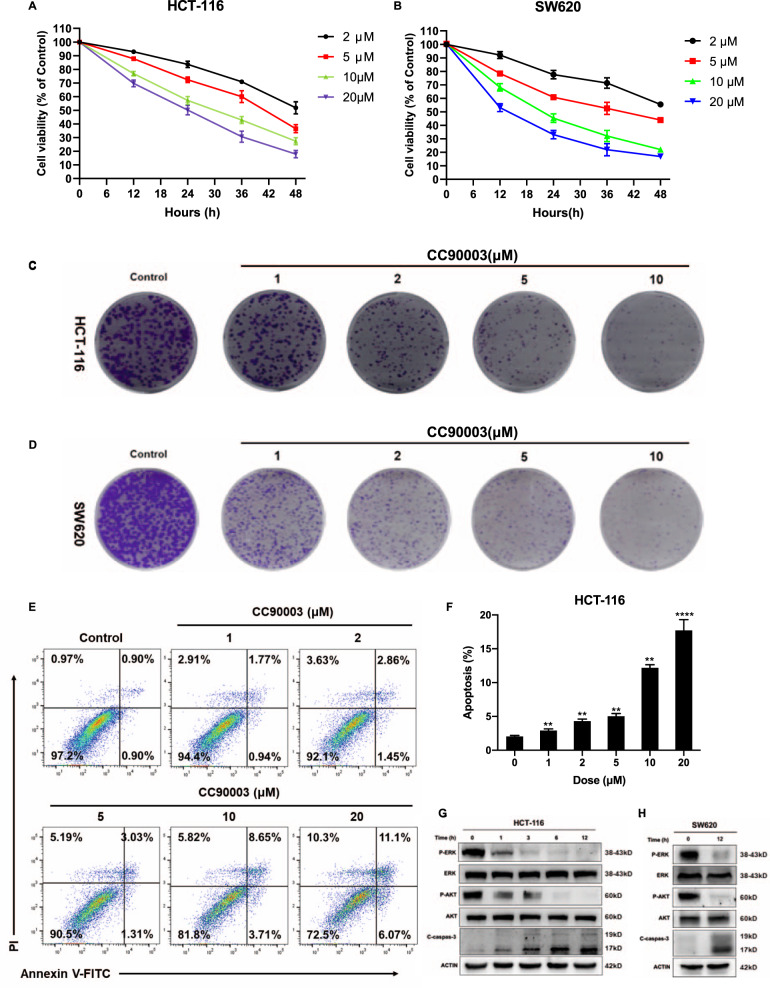
CC90003 affected cell death. **A**, **B** HCT-116 and SW620 cells were cultured at various does of CC90003 for 12, 24, 36, and 48 h, and then the cell viability was detected by the CCK-8 assay. **C**, **D** Clone Formation Images of HCT-116 and SW620 cells treated with different dose of CC90003 for 14 days. **E**, **F** Flow Cytometry was performed to detect cell apoptosis after treating with dose gradient of CC90003. Annexin-V-positive cells were defined as apoptosis. **G**, **H** HCT-116 cells were treated with 10 μM CC90003 for 1, 3, 6, and 12 h, and SW620 cells were treated for 12 h. Western blotting was used to detect the level of ERK, p-ERK, AKT, p-AKT, C-caspase-3, and ACTIN.

To explore the influence of CC90003 on CRC cells and establish a proper dose for our system, HCT-116 and SW620 cells were treated with various doses of CC90003. Cell viability was analyzed using Cell Counting Kit-8 after 0, 12, 24, 36, and 48 h’ treatment. Cell viabilities showed a decrease with increasing doses, and a correlation was observed with posttreatment period in both HCT-116 and SW620 cells (Fig. 1A, B). Similar results were obtained from colony formation assay (Fig. 1C, D). Next, to reveal the mechanism of CC90003-induced cell growth inhibition, flow cytometry was performed and the results showed that both apoptosis and nonapoptotic cell death occurred after CC90003 treatment (Fig. 1E, F). To further confirm our results, the activation of ERK and different markers of cell death were detected by western blotting. Figure 1G, H showed that the phosphorylation level of ERK and AKT decreased while the level of cleaved Caspase-3 increased markedly in both HCT-116 and SW620 cells, which indicated that CC90003 effectively inhibited ERK activation and induced colon cancer cell apoptosis. This apoptotic induction might be through AKT signaling pathway, which was demonstrated by our previous studies [23, 24]. In addition, GPX4 decreased while PAR increased obviously (Fig. S1A and B) suggested that ferroptosis and parthanatos might also be induced by CC90003. Together, targeting ERK suppressed colon cancer cell growth by inducing apoptosis as a primary minor, during which ferroptosis and parthanatos might play some roles.

### Targeting ERK could also induce autophagy

**Fig. 2:**
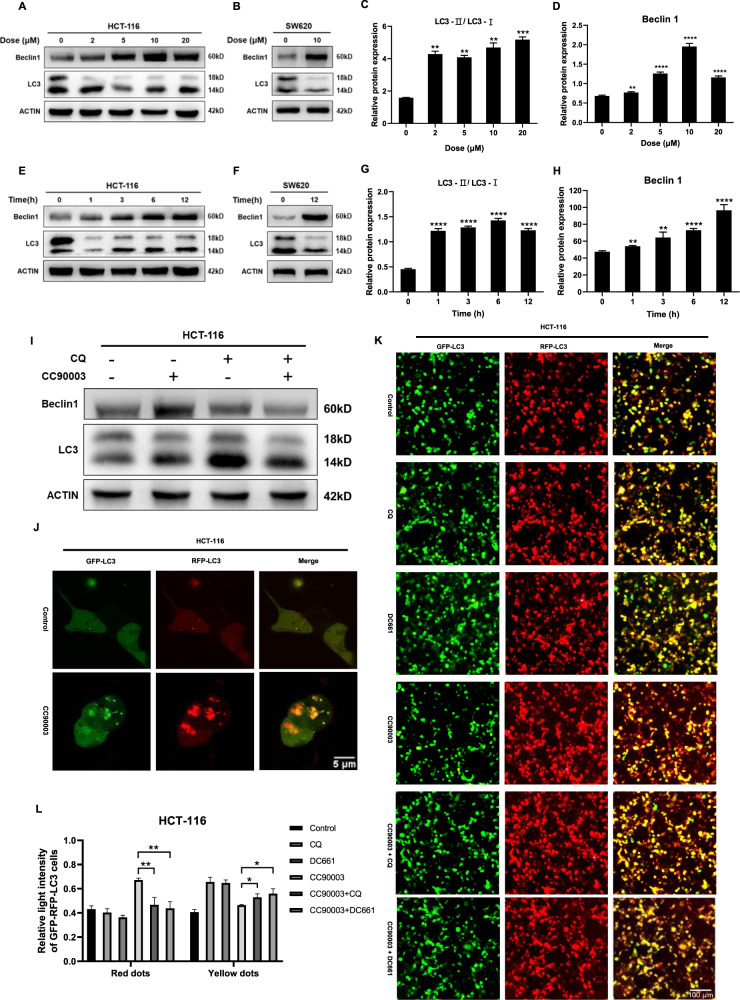
CC90003 induced autophagy in CRC cells. **A**–**D** Beclin1, LC3, and ACTIN proteins were detected by western blotting. HCT-116 cells were treated with various dose of CC90003 for 24 h, and SW620 cells were treated with 10 μM CC90003 for 24 h. **E**–**H** Beclin1, LC3, and ACTIN proteins were detected by western blotting. HCT-116 cells were treated with 10 μM CC90003 for different hours, and SW620 cells were treated for 12 h. **I** Western blotting was used to detect the level of Beclin1 and LC3 after 24 h of treatment with CC90003 (10 μM), CQ (20 μM), or their combination. **J** The HCT-116 cells stably expressing the GFP-RFP-LC3 fusion protein were treated with CC90003, and were observed with a confocal microscope (2000 times magnification). **K**, **L** HCT-116 cells stably expressing GFP-RFP-LC3 fusion protein were treat with DMSO, CQ (20 μM), DC661 (1 μM), CC90003 (10 μM), CC90003 + CQ, CC90003 + DC661. The results were observed with a fluorescence microscope and we quantified relative light intensity with ImageJ software.

To confirm whether CC90003 caused autophagy, we took different doses of CC90003 to treat with HCT-116 and SW620 cell for different time and detect the level of LC3. As a result, the level of Beclin1 and LC3 were in a dose and time-dependent manner (Fig. 2A–H). Besides, to further confirm the autophagic flux, CC90003 was used alone or in combination with CQ. The results indicated that when the cells were treated with CC90003 combined with CQ, LC3-II/LC3-I ratio was higher than that when CC90003 was used alone, while the level of Beclin1 was reduced (Fig. 2I). The above results showed that CC90003 could induce autophagy in CRC cells, which was dose- and time-dependent and could be inhibited by CQ. In addition, the GFP-RFP-LC3 plasmid was transfected into CRC cells. As shown in Fig. 2J, the fluorescence images showed that the number of yellow and red color puncta were more than the control group, meaning CC90003 induced autophagic flux in living cells. Moreover, CRC cells were treated with autophagy inhibitor CQ or DC661, the yellow spots increased and red spots decreased (Fig. 2K, L), which confirmed the results from western blotting assays that CC90003 could induce autophagy in CRC cells and this kind of autophagy could be inhibited by the autophagy inhibitors. Taken together, our data suggested that CC90003 induced autophagy in CRC cells, which might be suppressed by autophagy inhibitors.

### Inhibition of autophagy could increase cell death caused by CC90003

**Fig. 3:**
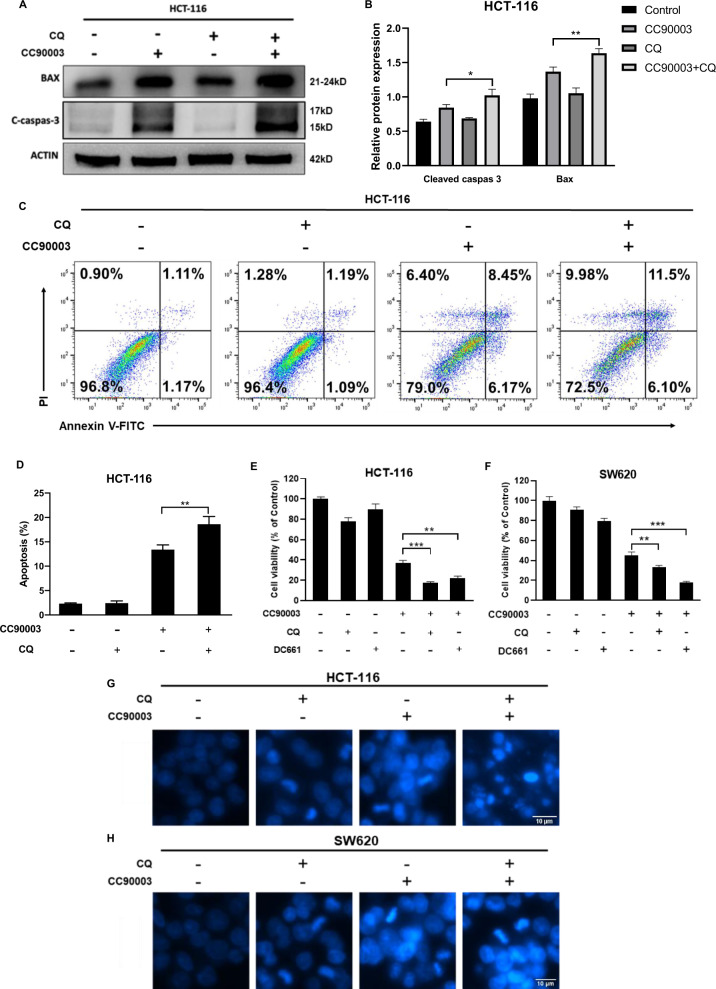
The relationship between autophagy and cell death. **A**, **B** Western blotting was performed in HCT-116 cells treated with CC90003 (10 μM) for 24 h in the absence or presence of CQ (20 μM). **C**, **D** AV/PI staining and flow cytometry analysis of apoptosis in HCT-116 cells treated with CC90003 (10 μM), CQ (20 μM), or their combination. Annexin-V-positive cells were defined as apoptosis. **E**, **F** HCT-116 cells and SW620 cells were cultured with DMSO, CQ (20 μM), DC661 (1 μM), CC90003 (10 μM), CC90003 + CQ, CC90003 + DC661 for 24 h, and then the cell viability was detected by the CCK-8 assay. **G**, **H** Hoechst33342 staining was carried out to examine the morphological changes of apoptosis in CRC cells treated with CC90003 (10 μM), CQ (20 μM), or their combination for 24 h.

Several independent experiments were used to evaluate the effect of CC90003 and combination with autophagy flux inhibitor on cell death. The level of Bax and C-caspase-3 significantly increased after the combination treatment of CC90003 and CQ, comparing with the single CC90003 treatment (Fig. 3A, B). The results collectively indicated that the autophagy inhibitor CQ could promote cell apoptosis induced by CC90003. At the same time, the results of cell flow cytometry were also consistent with the above results that the number of FITC-positive apoptotic cells was higher in the combined treatment group than that in the single CC90003 treatment group (Fig. 3C, D). In addition, the CCK-8 assay and Hoechst33342 staining were conducted in HCT-116 and SW620 cell with multiple treatments. The results showed that compared with single CC90003 treatment, the combined treatment significantly reduced cell viability and increased the death rate (Fig. 3E–H). Therefore, the combination therapy with CC90003 and CQ is more effective than the treatment with single substance in patients with tumors.

### ROS could mediate CC90003-induced autophagy and cell death

**Fig. 4:**
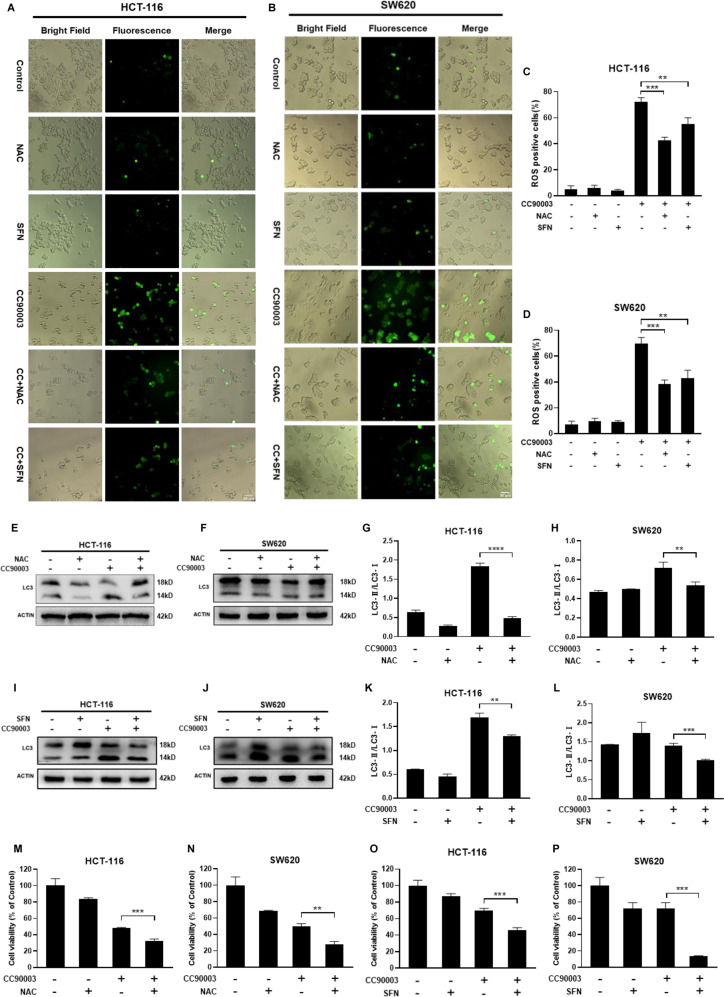
The relationship involved in ROS and autophagy. **A**–**D** The representative images of DCFH-DA fluorescence in HCT-116 and SW620 cells treated with CC90003 (10 μM), NAC (20 μM), SFN (20 μM), CC90003 + NAC, and CC90003 + SFN were observed by a fluorescence microscope [20x]. **E**–**L** Western blotting assay tested the level of LC3 in CRC cells after treating with CC90003 (10 μM), NAC (20 μM), SFN (20 μM), CC90003 + NAC, and CC90003 + SFN. **M**–**P** HCT-116 and SW620 cells were cultured with CC90003 (10 μM), NAC (20 μM), SFN(20 μM), or their combination for 24 h, and then the cell viability was detected by the CCK-8 assay.

To further verify how CC90003 promoted autophagy and cell death in CRC cells, the ROS generation was determined in HCT-116 and SW620 cells with ROS probe DCFH-DA. The fluorescence images indicated that CC90003 significantly increased the number of ROS-positive cells in HCT-116 and SW620 cells, and the number of ROS-positive cells decreased by two ROS inhibitor (Fig. 4A, B), which also indicated that the ROS production induced by CC90003 could be suppressed by the ROS inhibitors (Fig. 4A–D). And, another ERK inhibitor MK8353 was also used for experiments, and the same results were obtained (Fig. S2A, B). In addition, the western blotting assays performed in the two kinds of cells showed that the groups with combination treatment of CC90003 and ROS inhibitors had lower LC3-II/LC3-I ratios compared with that of the single CC90003 treatment groups. Besides, the CC90003 treatment combined with the ROS inhibitor NAC or SFN might inhibit the autophagy flux induced by CC90003 (Fig. 4E–L). Moreover, the CCK-8 assay showed that the inhibition of ROS had an enhanced effect on the cell viability decreased by CC90003 (Fig. 4M–P). Taken together, these results indicated that CC90003 induced autophagy and death of CRC cells in a ROS-dependent manner.

### P53 is crucial for CC90003-induced autophagy and cell death

**Fig. 5:**
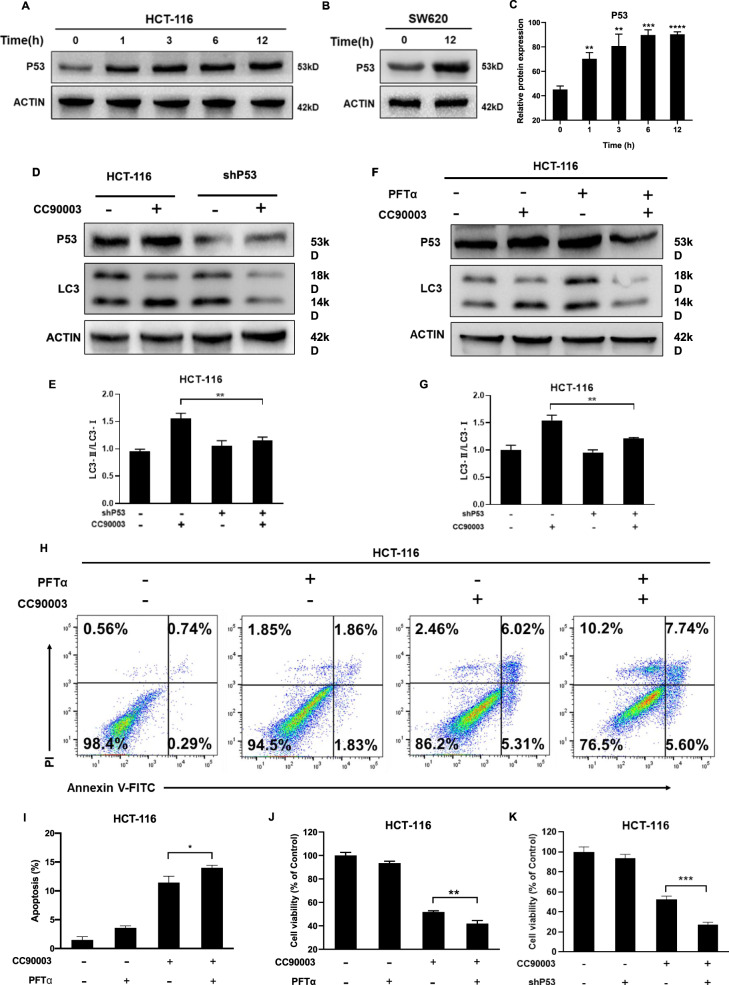
The relationship between P53 and autophagy. **A**–**C** HCT-116 cells were treated with 10 μM CC90003 for 1, 3, 6, 12 h, and SW620 cells were treated for 12 h. Western blotting was used to detect the level of P53 and ACTIN. **D**, **E** Western blotting showed the level of P53, LC3, and ACTIN in shP53 CRC cells after 10 μM CC90003 treatment. **F**, **G** Western blotting detected the level of P53, LC3, and ACTIN in CRC cells after treating with CC90003, PFTα, or their combination. **H**, **I** FITC/PI staining and flow cytometry analysis of apoptosis in HCT-116 cells treated with CC90003 (10 μM), PFTα (30 μM), or their combination. Annexin-V-positive cells were defined as apoptosis. **J**, **K** CRC cells were treated with CC90003 (10 μM), PFTα (30 μM), shP53, or their combination for 24 h, and then the cell viability was detected by the CCK-8 assay.

As shown in Fig. 5A–C, the western blotting analysis showed that the level of p53 were increased in a time-dependent manner. We investigated that whether silencing or inhibiting p53 affected autophagy and cell death caused by CC90003. When p53 is knocked down or inhibited by the p53 inhibitor pifithrin-α (PFTα), according to the changes of LC3-II/LC3-I ratios, the autophagy flux induced by CC90003 is significantly suppressed (Fig. 5D–G). In addition, we carried out cell flow cytometry and CCK-8 assay. As shown in Fig. 5H–K, the results showed that after the combined treatment of PFTα and CC90003, the proportion of apoptosis and the proportion of cell viability increased. In summary, p53 mediated the autophagy induced by CC90003, and the decreased level of p53 enhanced the cell death induced by CC90003.

### The combinational therapy with CQ could enhance the antitumor effect of CC90003 in vivo

**Fig. 6:**
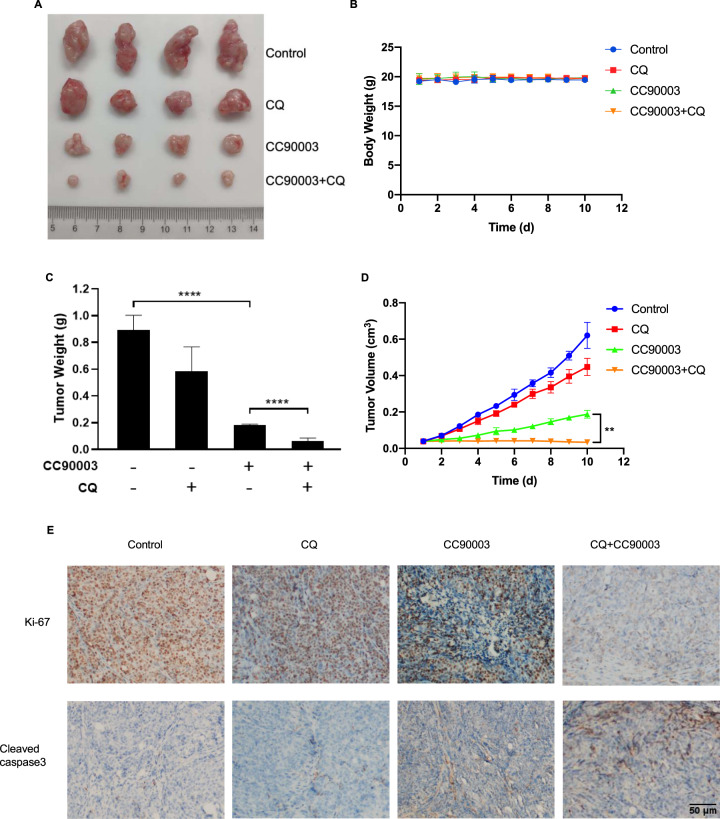
The antitumor effects of CC90003 and CQ in vivo. **A** Representative tumor images of the xenograft tumors. **B** Xenograft body weights were measured twice daily. **C** Average tumor weight was calculated. **D** Tumor volume at indicated time points after treatment was calculated. **E** IHC staining analyzed representative images of IHC staining of Ki-67 and cleaved-caspase-3.

**Fig. 7:**
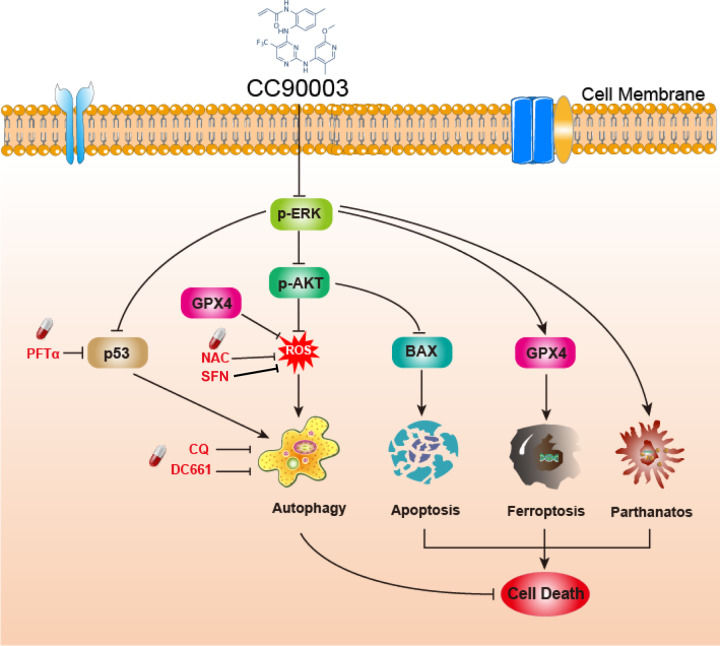
Schemes and mechanisms showed the whole process of the experiment. Targeting ERK leads to cell death and p53/ROS-dependent protective autophagy simultaneously.

According to the above results, the combination treatment of CC90003 and autophagy inhibitors had a stronger antitumor effect compared with the single CC90003 treatment. To confirm this conclusion, the nude mice subcutaneous tumor xenograft model was established in vivo. As shown in the Fig. 6A, the average tumor weight and volume in the CC90003-treated groups were significantly smaller than those of the control group, and the combined treatment group possessed the strongest antitumor effect. Moreover, immunohistochemistry was performed to detect the level of Ki-67 and cleaved-caspase-3 in xenograft tumors. Compared with the control group, after treated with CC90003, Ki-67-positive points were significantly reduced, and the cleaved-caspase-3-positive points were significantly increased in the tissues. And after the mixture of medication treatment, the effect of CC90003 was more obviously (Fig. 6E). Taken together, the above experimental results confirmed that CC90003 might inhibit the growth of CRC cells in vivo and this effect would be stronger after inhibiting autophagy. Additionally, the mechanism of the relationships between autophagy and cell death, and the roles of ROS found in the present study was showed in Fig. 7.

## Discussion

CRC is a common gastrointestinal tumor and the fourth leading cause of cancer death in the world [25]. Complete surgical resection, chemotherapy, and targeted therapy are the main treatments for CRC patients. However, due to the lack of specificity and the emergence of drug resistance, the effectiveness of the traditional chemotherapy drugs may be limited [26, 27]. Therefore, there is an urgent need to find new drugs to improve the effect of chemotherapy.

CC90003 is a novel type of ERK inhibitor. Studies have confirmed that this drug has a good antitumor effect in CRC cells [28]. However, its specific mechanism needs to be further explored. In this study, based on the preliminary experimental results, we knew that CC90003 could cause the death of CRC cells and induce apoptosis (Fig. 1), and there possibly existed other kinds of cell death caused by CC90003 (Fig. S1). Next, we found that CC90003 could significantly induce autophagy in CRC cells (Fig. 2). The role of autophagy in cancer cells is a double-edged sword, which can promote or inhibit the growth of cancer cells [29–31]. On the one hand, anticancer drugs-induced autophagy can protect cancer cells from chemotherapy, which results in drug resistance [32]. On the other hand, anticancer drugs-induced autophagy can also activate apoptosis signal pathways to increase cancer cell death [33]. Therefore, our research also focused on the autophagy regulation in CRC, especially the autophagy induced by the chemotherapy drugs. Furthermore, we found that p53 and ROS played a key role in this process, and we also conducted an in-depth discussion.

According to previous reports, it is still unclear about the relationship between ERK and autophagy. Targeting ERK could not only elevate autophagy [8, 9], but suppress autophagy [10]. And our results illustrated that autophagy was induced after ERK inhibition, which demonstrated that in this study, CC90003 upregulated autophagy in CRC cells. At present, studies have confirmed that there is a close but uncertain connection between autophagy and cell death [34–36]. For instance, both prosurvival [8, 11] and prodeath [12] autophagy could be induced by ERK inhibition. In our study, we treated CRC cells with the combination of CC90003 and autophagy inhibitor CQ and DC661. It was confirmed that CC90003 could enhance autophagy and cell death in CRC cells. And we conducted a variety of independent experiments and found that blocking the autophagy of CRC cells induced by CC90003 could increase the proportion of cell death, which showed that autophagy played a protective role in the process. The conclusions were also obtained in subsequent animal experiments. Therefore, it can be considered that in CRC cells, the autophagy inhibitor CQ can significantly enhance the antitumor effect of CC90003, which provides a good idea for the research of antitumor drug resistance.

Studies have also confirmed that ROS can be used as an important molecule in cells to regulate tumor cell death [17]. Lower concentrations of ROS can normally participate in intracellular signal transduction, while higher concentrations of ROS can damage intracellular proteins and DNA, leading to autophagy or cell death [37, 38]. In our research, we found that CC90003 induced the production of high concentrations of ROS. Another ERK inhibitor MK8353 was also used (Fig. S2), which resulted in the similar conclusion that ERK inhibition could increase the high production of ROS. According to the mainstream points, high production of ROS generally drives cells to death [39]. Hence we carried out experiments in depth to verify the role of CC90003-induced ROS. Two kinds of ROS inhibitor, NAC and SFN, were separately used to combine with CC90003 treatment. After inhibiting ROS, cell death increased, which seemed to be contrary to the mainstream point. To ascertain the cause, we simultaneously detected the levels of autophagy, which finally turned out to be decreased. The result confirmed that ROS was involved in the production of autophagy. After inhibiting ROS, the autophagy induced by CC90003 reduced, while cell death increased. Therefore, we confirmed that CC90003 could induce autophagy by activating ROS (Fig. 4).

In our research, we also found that the tumor suppressor p53 is involved in the regulation of autophagy and cell death (Fig. 5). P53 is a tumor suppressor protein, a regulator of cell cycle progression and apoptosis that had been widely studied. Studies have reported that HCT-116 cells that have been knocked out of p53 can improve the survival of mice by inducing autophagy [40], while retransfection with wtp53 can inhibit baseline autophagy [41]. On the contrary, some studies suggest that p53 can induce autophagy by upregulating AMPK [21, 42]. In our study, it was found that after silencing p53 or using the p53 inhibitor PFTα, the autophagy induced by CC90003 was weakened. In addition, the rate of cell death increases. These results indicate that p53 plays a role in inducing autophagy.

In general, a large number of studies have confirmed that inhibition of autophagy is considered to be a way to treat cancer, especially in combination with chemotherapy drugs [43]. In our study, it was also demonstrated that inhibiting autophagy could increase the death of CRC cells. The combination of CQ and CC90003 in the treatment of CRC had a strong additive antitumor effect (Fig. 6), and might be serve as a new strategy for the treatment of CRC. In addition, our results also confirmed that p53 and ROS were closely involved in the interaction process of autophagy and cell death, which will provide a basis for further research on the molecular mechanism of autophagy.

## Materials and methods

### Antibodies and reagents

Antibodies used in this study were listed in Supplementary Table S1. ECL-plus kit was from NCM Biotech in China. The Chloroquine (CQ) was purchased from Sigma–Aldrich Chemical Company (St. Louis, MO, USA). And CC90003, DC661, N-acetyl-L-cysteine (NAC), Sulforaphane (SFN) were obtained from Selleck Chemicals (Houston, TX, USA). Lipofectamine 2000 was purchased from Invitrogen (USA).

### Cell culture and treatments

Human CRC cell HCT-116 and SW620 were purchased from American Type Culture Collection (ATCC). The cells were cultured in DMEM medium (Gibco) routinely containing 10% fetal bovine serum (FBS), penicillin (100 units/mL), and streptomycin (100 mg/mL) in the humidified incubator.

### Cell viability assay and apoptosis assays

Cells were cultured in a 96-well microplate at a density of 5 × 10^3^ cells/well for 24 h. Then, the cells were divided into several groups and treated with different conditions. Cell viability was assessed with CCK-8 (Cell Counting Kit-8, 7 sea biotech, Shanghai, China) after treating drugs. The absorbance value at 450 nm (OD450) was read with a 96-well plate reader (DG5032, Huadong, Nanjing, China). Nuclear staining with Hoechst33342 (Invitrogen) was used to analyze the apoptosis. For colony formation assays, an equal number of cells after different treatments were planted in six-well plates. Colonies were visualized by crystal violet staining 14 days after plating.

### Flow cytometry

Cells were seeded in six-well plates and then treated with indicated dose of drugs for 24 h. After treatment, cells were harvested, washed with PBS, and resuspended by binding buffer. Then the cells were stained with Annexin-V-FITC and propidium iodide (PI) for 15 min at room temperature in the dark. Apoptosis was quantified using flow cytometry. Annexin-V-positive cells were defined as apoptotic cells.

### ROS detection and measurement

ROS induced by CC90003 was determined by using ROS Assay Kit (Beyotime, Shanghai, China). Briefly, after exposure to CC90003 for 24 h, the cells were incubated with 10 μM DCFH-DA in the dark for 20 min. The cells were then observed using a fluorescence microscope. Calculation of proportion of ROS-positive cells was used as result quantification.

### Western blotting

Equivalent protein samples from cells (30 μg protein extract was loaded on each lane) were subjected to the SDS-PAGE gel. The proteins were then transferred onto PVDF membranes (Millipore) and blocked with 5% non-fat milk for 90 min at room temperature. The membranes probed with the indicated primary antibodies were incubated at 4 °C overnight. Primary antibody was detected using horseradish peroxidase (HRP)-conjugated anti-rabbit or anti-mouse secondary antibody with an ECL-plus kit. Detection was performed using the Odyssey infrared imaging system (LI-COR, Lincoln, NE). Each western blotting was performed independently at least three times. The band intensity was quantified by ImageJ software and the data were normalized to actin level.

### Cell transfection

The GFP-RFP-LC3 plasmid and shRNA of p53 were purchased from Hansheng Technology. The plasmid or shRNA was transfected with Lipofectamine 2000 into cells following the manufacturer’s instructions. After 24 h, we renewed the medium. And after another 24 h, the transfected cells were treated according to the experiment plan.

### Xenograft mouse model and treatments

All animal experiments were carried out followed the protocols approved by Hunan University Animal Use and Care Committee (Changsha, Hunan, China; SYXK2018-0006). The xenograft mouse models carrying human CRC HCT-116 cells were established in 5- to 6-week-old female nude mice (Vital River, China). No blinding was done. Sixteen of mice were randomly divided into four groups. 1 × 10^6^ cells were resuspended in 100 μL of PBS and subcutaneously injected into the flanks of nude mice. Once the xenograft tumors reached 50–100 mm^3^, mice were treated daily with 25 mg/kg CC90003 by oral gavage and CQ at 25 mg/kg by i.injection, and their combination for 10 days. CC90003 was dissolved in NMP/PEG300 (1:9) and sonicated in NMP firstly. Then, PEG300 was added till the final volume. And CQ was supplied as a stock solution. Mice were terminated after 10 days of treatment. Tumor growth was monitored by calipers, and tumor volumes were calculated by the formula 0.5 × length × width^2^. Mice were euthanized when tumors reached ~1.0 cm^3^ in size.

### Histopathology

For immunohistochemistry (IHC), tumors were fastened in 4% paraformaldehyde for 24 h. After dehydration, tissues were embedded in paraffin and sectioned by LEICA system according to standard protocols. Briefly, 1% hydrogen peroxide was used to blocked antigen retrieval with citric acid (pH 6.0) endogenous peroxidase activity. Primary antibody was applied and tissues were incubated with secondary antibodies conjugated to peroxidase-labeled dextran polymer. Sections not exposed to secondary antibody were served as negative controls.

### Statistical analysis

Statistical analyses were carried out using the GraphPad Prism V software. All assays were repeated independently for a minimum of three times. Data were represented as mean ± SEM in the figures. *P-*values were calculated using the Student’s paired *t*-test. Differences were considered statistically significant at *p < 0.05, **p < 0.01, ***p < 0.001, ****p < 0.0001.

## Supplementary information

Figure S1

Figure S2

Supplementary legends

CC90003_Table S1

## Data Availability

The datasets generated and/or analyzed during the current study are available from the corresponding authors on reasonable request.
