# Supplementary figures and images for: Targeting ERK induced cell death and p53/ROS-dependent protective autophagy in colorectal cancer
Source: Cell Death Discov. 2021 Dec 4;7:375. doi: 10.1038/s41420-021-00677-9 (PMC8643355; doi:10.1038/s41420-021-00677-9)

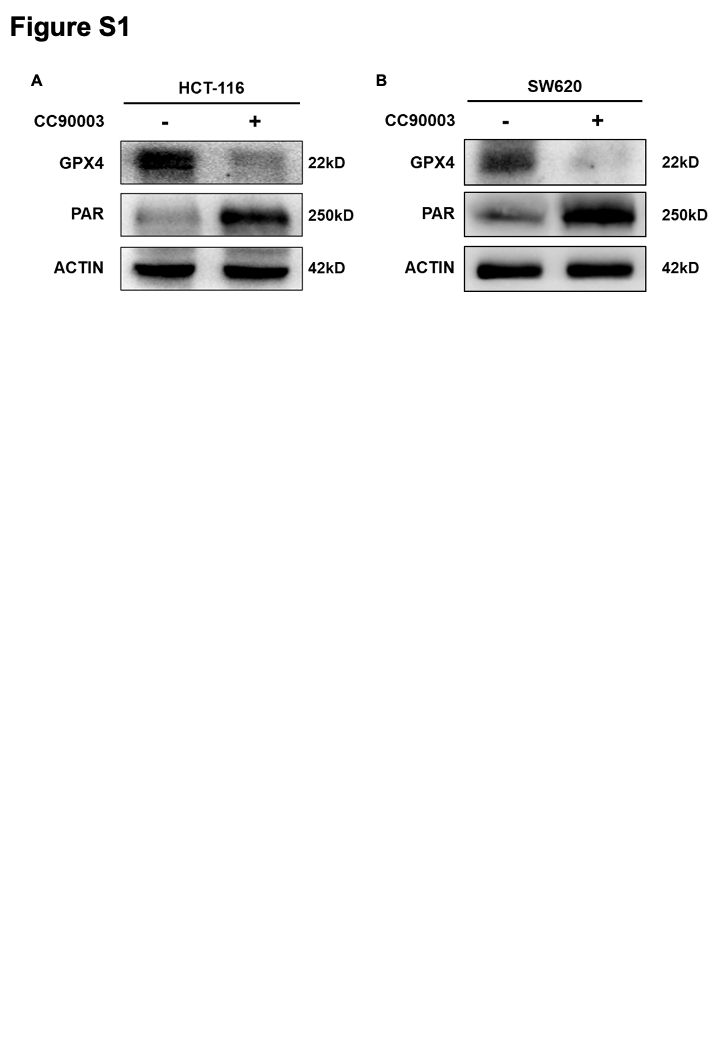

Supplement: Supplementary file 1 — Figure S1 [file 41420_2021_677_MOESM1_ESM.tif]

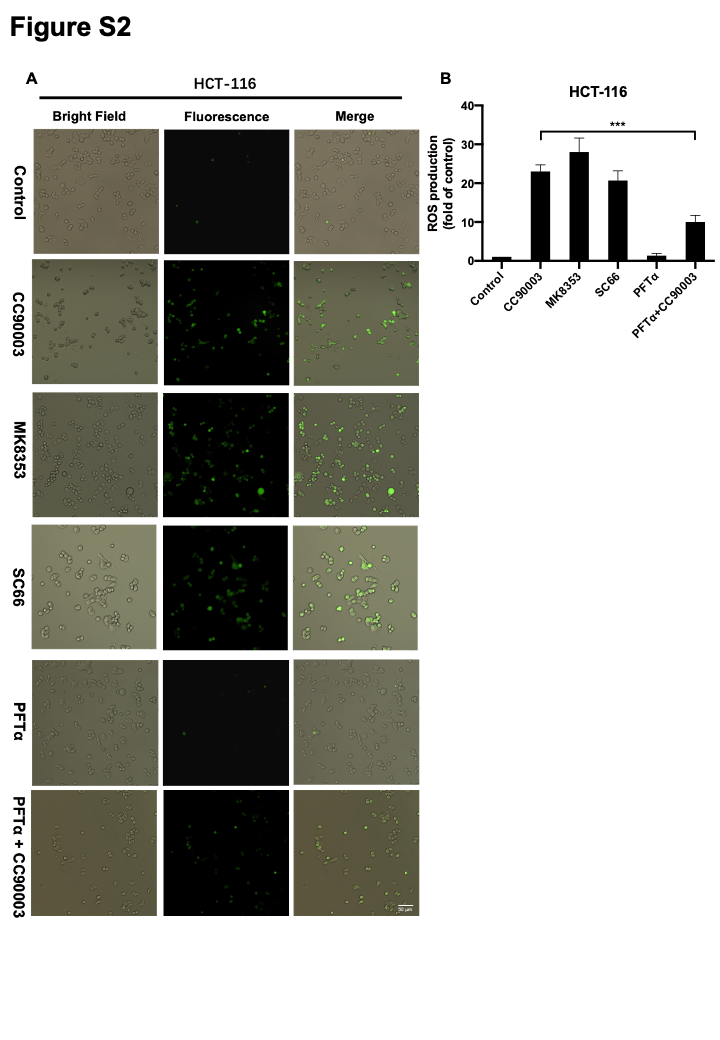

Supplement: Supplementary file 2 — Figure S2 [file 41420_2021_677_MOESM2_ESM.tif]
