# Supplementary material for: Targeting ERK induced cell death and p53/ROS-dependent protective autophagy in colorectal cancer
Source: Cell Death Discov. 2021 Dec 4;7:375. doi: 10.1038/s41420-021-00677-9 (PMC8643355; doi:10.1038/s41420-021-00677-9)
Supplement: Supplementary file 3 — Supplementary legends [file 41420_2021_677_MOESM3_ESM.docx]

**Supplemental materials legends**

**Fig.S1** CC90003 could induce more kinds of modes than apoptosis. **A** Western blotting was performed to detect GPX4 and PAR level in HCT-116 and SW620 cells after treating with 10 μM CC90003 for 24 h. **B** The statistical graph of A.

**Fig.S2** The antitumor effects of CC90003 and CQ in vivo. **A** The representative images of fluorescence intensity of DCFH-DA in HCT-116 cells treated with CC90003 (10 μM), MK8353 (10 μM), SC66 (10 μM), PFTα (30 μM), and CC90003+ PFTα observed by a fluorescence microscope (20x). Calculated the proportion of ROS positive cells for quantification. **B** The statistical graph of A.
